# Hahnemann’s Homœopathic Formula

**Published:** 1884-11

**Authors:** R. E. Dudgeon

**Affiliations:** London


### HAHNEMANN'S HOMŒOPATHIC FORMULA.

DR. R. E. DUDGEON, LONDON.

Dr. Lippe, in your September number, replying to my remarks in your July number, says: "Dr. Dudgeon * * * contends that the complete homœopathic formula should always read *curentur;* that *curantur* would be a blunder, because it would show that our formula (with *curantur* accepted) was an acknowledgment of 'a law,' while *curentur* would mean that we had only to do with a rule, good at times, but never to be accepted or applied imperatively."

Dr. Lippe must credit your readers with very short memories, that he ventures in September to ascribe to an article of mine published in July words and statements that I never made use of and that I entirely repudiate.

My little article, as perhaps your readers will remember, was in reply to Dr. Lippe's assertion that Hahnemann employed the formula "*similia similibus curantur.*" I showed that Hahnemann always wrote *curentur*, and never *curantur.* I then said: "If, then, *curentur* implies that the homœopathic formula is a therapeutic rule, whereas *curantur* implies that it is a law of nature, *as Dr. Lippe seems to assert*, then Hahnemann's use of *curantur* would show that his idea was that the formula merely expressed a therapeutic rule." By the words in italics it will be observed that the supposed material difference between *curentur* and *curantur* was Dr. Lippe's, not mine. For my own part, I don't think there is any important difference of meaning between the one phrase and the other. *Similia similibus curentur*, let likes be treated by likes, seems to be the more appropriate form for the promulgation of the new therapeutics; *similia similibus curantur*, likes are treated by likes, the most fitting expression in the mouths of the practitioners of the new system. So Priessnitz, the founder of the water-treatment, or *wassercur*, might have said, *Morbi aquâ curentur*, let diseases be treated by water, and his disciples might have inscribed over their establishments, *Morbi aquâ curantur*, diseases are treated by water.

I never allowed that *curantur* was wrong; all I did was to show that Dr. Lippe's assertion that it was the word used by Hahnemann was erroneous. The reappearance of the word on the cover of the *B. J. of H.* only shows the little importance the editors attach to the alternative readings, and not that we are "badly beaten," of which Dr. Lippe, whose ignorance of what Hahnemann actually wrote has just been exposed, will hardly be admitted to be a fit judge.

To say, as Dr. Lippe did, that Dr. Hughes, in adopting Hahnemann's own formula, *similia similibus curentur*, "has departed from Homœopathy hopelessly," is about as absurd a statement as could be made. If he has thereby departed from Homœopathy he has done so in very good company, that of Hahnemann himself. To imagine that the employment of one word or the other implies an adhesion to or a rejection of Homœopathy seems to originate in the notion that *similia similibus curantur* means, likes are cured by likes, a translation of the verb *curare* which would hardly occur to a "ripe classical scholar" (which Dr. Lippe says Hahnemann was), and would seem to be impossible to a German, whose words "*cur*" and "*curiren*" (derived from the Latin *curo*) are always used to signify *treatment* and *treat*, and are used by Hahnemann in that and in no other sense in many places.

I can easily understand that Dr. Lippe, who has always posed as a Hahnemannian of the purest water, should be very much annoyed at being shown to be unaware that Hahnemann used *curentur*, and not *curantur*, as the formula of his therapeutics, especially as he made Hahnemann's supposed use of *curantur* the ground of an attack on Dr. Hughes; but I do not think that his annoyance justifies him in misrepresenting what I said, and in making use of such discourteous language as that he thinks fit to indulge in.

Surely, members of a liberal profession, ostensibly pursuing the same object, might respect the courtesies and decencies of literary controversy in their discussions. But what is to be said for Dr. Lippe, who, because I have shown him to be ignorant of a word employed by our common master, does not hesitate to deface your pages by accusations and insinuations against the *British Journal of Homœopathy* and its editors which I will not condescend to repeat, and endeavors to hide his own discomfiture by indulging in ponderous and pointless jokes which can only raise the laugh against himself?

